# Association between anxiety, depression, and symptom burden in patients with advanced colorectal cancer: A multicenter cross‐sectional study

**DOI:** 10.1002/cam4.7330

**Published:** 2024-06-07

**Authors:** Lili Song, Zhongge Su, Yi He, Ying Pang, Yuhe Zhou, Yu Wang, Yongkui Lu, Yu Jiang, Xinkun Han, Lihua Song, Liping Wang, Zimeng Li, Xiaojun Lv, Yan Wang, Juntao Yao, Xiaohong Liu, Xiaoyi Zhou, Shuangzhi He, Yening Zhang, Jinjiang Li, Bingmei Wang, Lili Tang

**Affiliations:** ^1^ Department of Psycho‐Oncology, Key Laboratory of Carcinogenesis and Translational Research (Ministry of Education/Beijing) Peking University Cancer Hospital & Institute Beijing China; ^2^ Department of Breast Cancer Radiotherapy, Chinese Academy of Medical Sciences Cancer Hospital Affiliated to Shanxi Medical University Taiyuan China; ^3^ The Fifth Department of Chemotherapy The Affiliated Cancer Hospital of Guangxi Medical University Nanning Guangxi China; ^4^ Department of Medical Oncology, Cancer Center, West China Hospital Sichuan University Chengdu China; ^5^ Department of Breast Medical Oncology Shandong Cancer Hospital and Institute, Shandong First Medical University and Shandong Academy of Medical Sciences Jinan China; ^6^ Department of Oncology The First Affiliated Hospital, Zhengzhou University Zhengzhou China; ^7^ Department of Oncology Xiamen Humanity Hospital Xiamen China; ^8^ Department of Integrated Chinese and Western Medicine Shaanxi Provincial, Cancer Hospital Affiliated to Medical College of Xi'an Jiaotong University Xi'an China; ^9^ Department of Clinical Spiritual Care Hunan Cancer Hospital/The Affiliated Cancer Hospital of Xiangya School of Medicine, Central South University Changsha China; ^10^ Radiotherapy Center, Hubei Cancer Hospital Wuhan China; ^11^ Department of Psycho‐oncology Shandong Cancer Hospital and Institute, Shandong First Medical University and Shandong Academy of Medical Sciences Jinan China

**Keywords:** advanced cancer, anxiety, colorectal cancer, depression, symptom burden

## Abstract

**Objectives:**

Patients with advanced colorectal cancer (CRC) have multiple concurrent physical and psychological symptoms. This study aimed to explore the relationship between anxiety, depression, and symptom burden in advanced CRC.

**Methods:**

A multicenter cross‐sectional study was conducted in 10 cancer centers from geographically and economically diverse sites in China. A total of 454 patients with advanced CRC completed the Hospital Anxiety and Depression Scale and the MD Anderson Symptom Inventory. Multiple regression analysis was applied to explore the relationship between anxiety, depression and symptom burden.

**Results:**

About one‐third of the patients showed symptoms of anxiety or depression. Patients with anxiety or depression reported significantly higher symptom burden than those without (*p* < 0.001). Patients with anxiety or depression reported a higher proportion of moderate‐to‐severe (MS) symptom number than those without (*p* < 0.001). About 52% of the patients with anxiety or depression reported at least three MS symptoms. The prevalence of MS symptoms was ranging from 7.3% (shortness of breath) to 22% (disturbed sleep), and in patients with anxiety or depression was 2–10 times higher than in those without (*p* < 0.001). Disease stage (*β* = −2.55, *p =* 0.003), anxiety (*β* = 15.33, *p* < 0.001), and depression (*β* = 13.63, *p* < 0.001) were associated with higher symptom burden.

**Conclusions:**

Anxiety and depression in patients with advanced cancer correlated with higher symptom burden. Findings may lead oncology professionals to pay more attention to unrecognized and untreated psychological symptoms in symptom management for advanced cancer patients.

## 1 INTRODUCTION

Colorectal cancer (CRC) is the third most common cancer and the second main cause of global cancer‐related mortality, and the incidence and mortality of CRC in China continue to rise higher than the world average rate.^1^ CRC patients may experience various physical and psychological symptoms due to their illness or treatment.^2^ Patients with CRC are at increased risk for anxiety and depression symptoms or disorders, which decreased quality of life and increased mortality in CRC patients.^3^, ^4^ About 20%–36.2% of patients with CRC had anxiety symptoms and 19%–30.1% of them had depression symptoms.^5^, ^6^ Almost 27.5% of advanced CRC patients suffer from anxiety symptoms as well as 28.3% of them suffer from depression symptoms.^7^

Most of the previous studies have focused on the relationship between psychological symptoms and one or several physical symptoms in patients with advanced cancer, such as pain, fatigue, and insomnia.^8^, ^9^, ^10^ Few studies have explored the relationship between psychological symptoms and overall symptom burden. To our knowledge, only two studies have examined the relationship between depression symptoms and physical symptom burden. Fitzgerald et al. found that depression symptoms were an independent predictor of physical symptom burden in patients with advanced cancer.^11^ Grotmol et al.'s study showed that patients with self‐reported depression disorder reported a twofold higher physical symptom burden compared with those without.^12^ Therefore, the relationship between anxiety symptoms and symptom burden in advanced cancer patients is still unclear.

Symptom burden is a significant cause of suffering in advanced cancer patients.^13^ Advanced CRC is related to high symptom burden and usually requires hospitalization.^14^ Our previous study showed that hospitalized patients with advanced cancer in China have multiple concurrent symptoms, but lack sufficient management.^15^ Good symptom management can improve the quality of life of cancer patients, and even prolong their survival.^16^ However, despite these benefits, psychological symptoms are often not recognized and treated, highlighting the need for further improvement in care.^17^

This study aimed to explore the relationship between anxiety, depression, and symptom burden in advanced CRC. We hypothesized that anxiety and depression symptoms are associated with symptom burden, assuming that patients with anxiety or depression have a higher symptom burden than patients without based on our clinical experience.

## 2 METHODS

### 2.1 Study design

This secondary analysis was part of a multicenter cross‐sectional study (Chinese Clinical Trial Registry: ChiCTR1900024957), which was conducted from August 2019 to December 2020.^15^ The last CRC patient was enrolled at December 20, 2019. To recruit a representative sample of cancer patients in China, 10 cancer centers were selected from different cities across China: Beijing, Shandong, Fujian, Guangxi, Hubei, Hunan, Henan, Shanxi, Shaanxi, and Sichuan. These cancer centers were selected based on diverse geographical location and level of economic development, which represent diverse socioeconomic status patients.

### 2.2 Participants

Eligible patients were 18 years of age or older, diagnosed with the advanced CRC, hospitalized for anticancer treatment and/or palliative care, and able to give informed consent. Patients were excluded if they had severe cognitive impairment or communication difficulties or were physically and mentally unable to finish the questionnaire independently. The study was approved by the Institutional Review Board of Peking University Cancer Hospital (2019YJZ34). Written informed consent was obtained from all patients.

### 2.3 Procedures

Inpatients from 10 cancer centers who met the inclusion criteria were invited to join in the study by their oncologists. Questionnaire were administered to patients by an electronic patient‐reported outcome platform. After patients completed the questionnaires, the data was uploaded to the platform in real time. The system platform converts the data into a standard data schema for data analysis. Our previous studies have shown that using the platform in patients with advanced cancer is time‐saving, energy‐saving, and effective.^18^

### 2.4 Measurements

Demographic variables included age, gender, marital status, and educational status. Clinical characteristics included the disease stage, the disease status, prior chemotherapy, prior radiotherapy, prior surgery, current cancer therapy, current drug therapy for symptoms, and current psychiatric drug therapy. And the Eastern Cooperative Oncology Group performance status, anxiety, depression, and symptom burden were assessed.

The Hospital Anxiety and Depression Scale (HADS) was used to assess anxiety and depression in the past week.^19^ It is generally used for patients with psychological symptoms in general hospitals and is also widely used for cancer patients. The Chinese version of the HADS has good reliability and validity.^20^ It contains 14 items including two subscales: Anxiety (HADS‐A) and Depression (HADS‐D). Both subscales range from 0 to 21 comprising seven items. A score of 9 or above indicates possible anxiety or depression.^20^

The Chinese version of the MD Anderson Symptom Inventory (MDASI‐C) was used to assess cancer‐related symptom burden.^21^ Symptom burden is defined as “having at least one moderate‐to‐severe (MS) symptom” among the core symptoms of MDASI.^15^ The MDASI‐C has good reliability and validity.^21^ It includes 13 symptom items. The MDASI‐C uses an 11‐point numeric scale ranging from 0 (nothing) to 10 (most severe). Scores of 5–6 indicate moderate symptoms, and 7–10 indicate severe symptoms.^21^ In the current study, symptom burden was calculated by the summing scores of the 13 symptoms on MDASI‐C.

### 2.5 Statistical analysis

Continuous variables were reported as the mean ± standard deviation. Categorical variables were reported as percentages (frequency). The cut‐off points of anxiety/depression were 9 as highlighted in the section “Measurements”. Differences in general characteristics, symptom burden and symptoms among patients with or without anxiety and depression were compared by using chi‐square tests for categorical variables, the independent *t*‐tests for continuous variables, respectively.

Patients were grouped by the number of reported MS symptoms into four groups based on previous studies^15^: no symptom, at least one MS symptom, at least three MS symptoms, and at least five MS symptoms. Differences in the number of MS symptoms among four groups were analyzed using chi‐square tests.

Multiple linear regression model was used to assess the relationship between symptoms burden and anxiety or depression adjusting for the covariates (age and gender) that we defined based on the literature. The variables associated with symptom burden in the univariate analyses were picked for the multiple linear regression analysis, in which symptom burden was used as a continuous variable. The dependent variable of multiple regression model was symptom burden and the independent variables (predictors) were anxiety, depression, and other clinical variables. A two‐sided *p* < 0.05 was considered statistically significant. All statistical analyses were performed using SPSS version 24.0 (SPSS Inc., Chicago, IL, USA).

## 3 RESULTS

### 3.1 Participant characteristics

A total of 455 patients with CRC were included in the study. After excluding one patient who did not complete all questionnaires that required for this study, there were 454 patients were entered into the analysis. The sample of 454 patients had a mean age of 56.9 years (standard deviation [SD] = 11.3; range = 19–87 years), and 58.1% were male. Participants were primarily married (95.6%). 53.5% of the patients were in the metastatic disease stage, and 44.3% were in stable disease status. Before hospitalization, 56.4% of patients received chemotherapy, 52.2% patients underwent surgery, and 12.6% received radiotherapy. After hospitalization, 59.7% of patients received anticancer therapy, 40.3% received palliative care, 25.3% received medication for symptom management, and 2.6% received psychiatric medication. The mean scores of symptom burden were 24.82 ± 21.07 (Table 1).

**TABLE 1:** Characteristics of the participants.

Characteristic	*N* (%)
Total, *n*	454
Age, mean (SD), years	56.9 (11.3)
Gender	
Female	190 (41.9)
Male	264 (58.1)
Marital status	
Married	434 (95.6)
Single, divorced or windowed	20 (4.4)
Education	
≤9 years	227 (50.0)
≥10 years	227 (50.0)
ECOG PS	
0	140 (30.8)
1	228 (50.2)
2	86 (18.9)
Disease stage	
No evidence of disease	20 (4.4)
Locoregional	180 (39.6)
Locoregional plus metastatic	11 (2.4)
Metastatic	243 (53.5)
Disease status	
Complete remission	16 (3.5)
Partial remission	104 (22.9)
Stable disease	201 (44.3)
Progressive disease	93 (20.5)
Unclear	40 (8.8)
Prior chemotherapy	
No	198 (43.6)
Yes	256 (56.4)
Prior radiotherapy	
No	397 (87.4)
Yes	57 (12.6)
Prior surgery	
No	201 (47.8)
Yes	237 (52.2)
Current cancer therapy	
Palliative care	183 (40.3)
Anticancer therapy	271 (59.7)
Current drug therapy for symptoms	
No	339 (74.7)
Yes	115 (25.3)
Current psychiatric drug	
No	442 (97.4)
Yes	12 (2.6)
Anxiety	
No	335 (73.8)
Yes	119 (26.2)
Depression	
No	317 (69.8)
Yes	137 (30.2)
Anxiety, mean (SD)	5.60 (4.09)
Depression, mean (SD)	5.92 (4.02)
Symptom burden, mean (SD)	24.82 (21.07)

Abbreviations: ECOG PS, Eastern Cooperative Oncology Group performance status; SD, standard deviation.

### 3.2 Anxiety and depression

As shown in Table 1, 119 patients (26.2%) had anxiety (HADS‐A ≥9); 137 patients (30.2%) had depression (HADS‐D ≥9). For total sample, mean scores of anxiety and depression were 5.60 ± 4.09 and 5.92 ± 4.02, respectively (Table 1). There were statistically significant differences between patients with depression or anxiety and those without depression or anxiety in terms of disease stage, disease status, and current cancer therapy (*p* < 0.05). There is a higher prevalence of anxiety (52.1% vs. 47.9%, *p =* 0.002), and depression (56.9% vs. 43.1%, *p* < 0.001) in patients receiving palliative care compared to those receiving anticancer therapy (Table 2).

**TABLE 2:** Univariate analysis for anxiety and depression.

Variables	Anxiety *n* (%)		*p*‐Value	Depression *n* (%)		*p*‐Value
	No	Yes		No	Yes	
Total, *n*	335 (73.8)	119 (26.2)		317 (69.8)	137 (30.2)	
Age,^a^ years	57.5 (11.3)	55.2 (11.4)	0.059	57.2 (11.4)	56.2 (11.3)	0.420
Gender						
Female	138 (41.2)	52 (43.7)	0.634	133 (42.0)	57 (41.6)	
Male	197 (58.8)	67 (56.3)		184 (58.0)	80 (58.4)	
Marital status						
Married	321 (95.8)	113 (95.0)	0.694	306 (96.5)	128 (93.4)	0.140
Single^b^	14 (4.2)	6 (5.0)		11 (3.5)	9 (6.6)	
Education						
≤9 years	170 (50.7)	57 (47.9)	0.594	157 (49.5)	70 (51.1)	0.759
≥10 years	165 (49.3)	62 (52.1)		160 (50.5)	67 (48.9)	
ECOG PS^c^						
0 and 1	277 (82.7)	91 (76.5)	0.137	264 (83.3)	104 (75.9)	0.066
2	58 (17.3)	28 (23.5)		53 (16.7)	33 (24.1)	
Disease stage						
No evidence of disease	13 (3.9)	7 (5.9)	<0.001	13 (4.1)	7 (5.1)	<0.001
Locoregional	108 (32.2)	72 (60.5)		102 (32.2)	78 (56.9)	
Locoregional plus metastatic	7 (2.1)	4 (3.4)		8 (2.5)	3 (2.2)	
Metastatic	207 (61.8)	36 (30.3)		194 (61.2)	49 (35.8)	
Disease status						
Complete remission	6 (1.8)	10 (8.4)	<0.001	5 (1.6)	11 (8.0)	<0.001
Partial remission	60 (17.9)	44 (37.0)		57 (18.0)	47 (34.3)	
Stable disease	165 (49.3)	36 (30.3)		162 (51.1)	39 (28.5)	
Progressive disease	71 (21.2)	22 (18.5)		62 (19.6)	31 (22.6)	
Unclear	33 (9.9)	7 (5.9)		31 (9.8)	9 (6.6)	
Current cancer therapy						
Palliative care	121 (36.1)	62 (52.1)	0.002	105 (33.1)	78 (56.9)	<0.001
Anticancer therapy	214 (63.9)	57 (47.9)		212 (66.9)	59 (43.1)	
Symptom burden^a^	18.29 (16.32)	43.20 (22.10)	<0.001	17.85 (16.55)	40.95 (21.61)	<0.001

*Note*: Significance level at *p* < 0.05.

^a^ Using mean (SD).

^b^ Including divorced or windowed.

^c^ For Eastern Cooperative Oncology Group performance status.

### 3.3 Symptom burden

Symptom burden was shown in Tables 2, 3, 4. Symptom burden scores is significantly higher in patients with anxiety (43.2 vs. 18.29, *p* < 0.001) and depression (40.95 vs. 17.85, *p* < 0.001) than those without (Table 2).

Compared to patients without anxiety (*n* = 335), patients with anxiety (*n* = 119) reported a higher proportion of MS symptom number (*p* < 0.001), over one symptom (70.6% vs. 41.8%), over three MS symptoms (52.9% vs. 19.4%), and over five MS symptoms (35.3% vs. 8.7%) (Table 3). Compared to the patients without depression (*n* = 317), patients with depression (*n* = 137) reported a higher proportion of MS symptom number (*p* < 0.001), over one MS symptom (68.6% vs. 41.0%), over three MS symptoms (52.6% vs. 17.7%), and over five MS symptoms (35.0% vs. 7.3%) (Table 3).

**TABLE 3:** Number of MS symptoms reported in patients with or without anxiety and depression.

No. of physical MS symptoms	Anxiety *n* (%)		*p*‐Value	Depression *n* (%)		*p*‐Value
	No (*n* =335)	Yes (*n* =119)		No (*n* =317)	Yes (*n* =137)	
0	195 (58.2)	35 (29.4)		187 (59.0)	43 (31.4)	
≥1	140 (41.8)	84 (70.6)	<0.001	130 (41.0)	94 (68.6)	<0.001
≥3	65 (19.4)	63 (52.9)	<0.001	56 (17.7)	72 (52.6)	<0.001
≥5	29 (8.7)	42 (35.3)	<0.001	23 (7.3)	48 (35.0)	<0.001

*Note*: Significance level at *p* < 0.05.

Abbreviation: MS, moderate‐to‐severe.

The prevalence of MS symptoms was ranging from 7.3% (shortness of breath) to 22% (disturbed sleep) in total sample. Disturbed sleep was the most prevalent symptom (22%), followed by fatigue (21.4%), dry mouth (19.8%), pain (17.4%), and lack of appetite (16.7%) among 13 symptoms (Table 4).

**TABLE 4:** Prevalence of symptoms in patients with or without anxiety and depression.

Variables^a^	Total sample *n* (%)	Anxiety *n* (%)		*p*‐Value	Depression *n* (%)		*p*‐Value
		No *n* = 335	Yes *n* = 119		No *n* = 317	Yes *n* = 137	
Fatigue	97 (21.4)	50 (14.9)	47 (39.5)	<0.001	48 (15.1)	49 (35.8)	<0.001
Upset	73 (16.1)	30 (9.0)	43 (36.1)	<0.001	25 (7.9)	48 (35.0)	<0.001
Disturbed sleep	100 (22.0)	58 (17.3)	42 (35.3)	<0.001	49 (15.5)	51 (37.2)	<0.001
Dry mouth	90 (19.8)	50 (14.9)	40 (33.6)	<0.001	50 (15.8)	40 (29.2)	0.001
Pain	79 (17.4)	42 (12.5)	37 (31.1)	<0.001	37 (11.7)	42 (30.7)	<0.001
Sad	56 (12.3)	20 (6.0)	36 (30.3)	<0.001	17 (5.4)	39 (28.5)	<0.001
Drowsiness	61 (13.4)	26 (7.8)	35 (29.4)	<0.001	25 (7.9)	36 (26.3)	<0.001
Lack of appetite	76 (16.7)	46 (13.7)	30 (25.2)	0.004	38 (12.0)	38 (27.7)	<0.001
Numbness or tingling	65 (14.3)	38 (11.3)	27 (22.7)	0.002	33 (10.4)	32 (23.4)	<0.001
Difficulty remembering	56 (12.3)	22 (6.6)	34 (28.6)	<0.001	27 (8.5)	29 (21.2)	<0.001
Nausea	56 (12.3)	27 (8.1)	29 (24.4)	<0.001	26 (8.2)	30 (21.9)	<0.001
Vomiting	40 (8.8)	20 (6.0)	20 (16.8)	<0.001	15 (4.7)	25 (18.2)	<0.001
Shortness of breath	33 (7.3)	7 (2.1)	26 (21.8)	<0.001	7 (2.2)	26 (19.0)	<0.001

*Note*: Significance level at *p* < 0.05.

^a^ For 13 moderate‐to‐severe (≥5) symptoms on the Chinese version of the MD Anderson Symptom Inventory (MDASI‐C).

In univariate analysis, patients with anxiety reported a higher prevalence of MS symptoms compared to those without anxiety (all *p* < 0.001), such as fatigue (39.5% vs. 14.9%), upset (36.1% vs. 9.0%), disturbed sleep (35.3% vs. 17.3%), dry mouth (33.6% vs. 14.9%), pain (31.1% vs. 12.5%), and sad (30.3% vs. 6.0%). Patients with depression reported a higher prevalence of MS symptoms compared to those without depression (all *p* **≤** 0.001), such as disturbed sleep (37.2% vs. 15.5%), fatigue (35.8% vs. 15.1%), upset (35.0% vs. 7.9%), pain (30.7% vs. 11.7%), dry mouth (29.2% vs. 15.8%), and sad (28.5% vs. 5.4%) (Table 4).

### 3.4 The relationship between anxiety, depression, and symptom burden

Multiple regression analysis showed that symptom burden could be predicted by the disease stage (*β* = −2.55, *p =* 0.003), anxiety (*β* = 15.33, *p* < 0.001) or depression (*β* = 13.63, *p* < 0.001) (Table 5).

**TABLE 5:** Multiple regression analyses of the relationship between anxiety, depression, and symptom burden.

Predictor variable	B	SE	Beta coefficients	*p*‐Value	95% CI
Dependent variable: symptom burden: adjusted *R* ^2^ = 0.349; *F* = 34.220, *p* < 0.001					
Age	0.03	0.07	0.02	0.700	−0.11 to 0.17
Gender	2.34	1.65	0.06	0.157	−0.90 to 5.58
Disease stage	−2.55	0.85	−0.13	0.003	−4.21 to −0.88
Disease status	0.26	0.89	0.01	0.772	−1.50 to 2.01
Current cancer therapy	1.03	1.73	0.02	0.550	−2.36 to −4.43
Anxiety	15.33	2.30	0.32	<0.001	10.81 to 19.84
Depression	13.63	2.19	0.30	<0.001	9.33 to 17.92

*Note*: Significance level at *p* < 0.05.

Abbreviations: CI, confidence interval; SE, standard error.

## 4 DISCUSSION

To the best of our knowledge, our study is the first to explore the relationship between anxiety, depression, and symptom burden in patients with advanced CRC. The most important finding of our study was that patients with anxiety or depression reported significantly higher symptom burden than those without.

The present study showed that 26.2% of patients with advanced CRC had symptoms of anxiety, and 30.2% of them had symptoms of depression, consistent with previous studies in advanced CRC^7^ and early postoperative CRC.^6^ These prevalence rates were significantly higher than those reported in previous studies in CRC survivors.^5^ The prevalence of MS symptoms ranged from 7.3% to 22% in patients with advanced CRC similar to previous studies,^22^ and in patients with anxiety or depression was 2–10 times higher than those without. For example, the prevalence of symptoms such as fatigue, disturbed sleep, pain, and drowsiness increased more than twice in patients with anxiety and depression. These findings suggest that patients with advanced CRC have high level of anxiety or depression and symptom burden.

Previous studies found cancer patients with depression had a higher symptom burden than non‐depressed patients.^11^, ^12^ Our study added that patients with advanced cancer and anxiety suffered from a higher symptom burden. Over 50% of patients with anxiety or depression suffered at least three MS symptoms, and nearly a third of patients with anxiety or depression had more than five MS symptoms. In the analysis of specific symptoms, the prevalence of each symptom is higher in patients with anxiety or depression than those without. Our study further explored that anxiety and depression were the predictors of symptom burden on multiple regression analyses. Compared to the study by Fitzgerald et al.,^11^ our study added a new finding that anxiety is also a predictor of symptom burden in advanced cancer.

Disturbed sleep, fatigue, dry‐mouth, pain and lack of appetite were reported as the top five MS symptoms in patients with advanced CRC, and the prevalence of these symptoms was more than twice higher in patients with anxiety or depression than those without. Cheng et al. found mild to moderate inter‐correlations among insomnia, fatigue, pain, and mood disturbance in cancer patients.^8^ It has been suggested that a depression‐pain‐fatigue symptom cluster,^23^, ^24^ a depression‐sleep‐pain symptom cluster^24^ or a sleep‐anxiety‐drowsiness symptom cluster^25^ may exist in advanced cancer. However, in addition to the symptoms in these symptom clusters, the prevalence of other physical symptoms such as nausea, vomiting, and shortness of breath were also significantly higher in patients with anxiety or depression than those without. These findings indicate that a collaborative relationship exists between anxiety, depression and physical symptoms in patients with advanced cancer.

Therefore, in clinical practice, the reciprocal effects of anxiety, depression, and symptom burden should be considered, so interventions on either one could impact positively the other one. It means that symptom management could improve patients' depression^26^ conversely interventions on anxiety and depression could reduce symptom burden among cancer patients.^27^

Unfortunately, there were a huge gap between the need of symptom management and received treatment in patients with advanced cancer. Only 25% of patients were undertaking drug therapy for their symptoms, and only 2.6% of the patients were taking psychiatric medication. It is consistent with the founding of Driessen et al. that the domain of psychological issues is the most unmet care need in advanced cancer patients.^17^ The low use of drug therapy for symptoms reveals the current clinical reality that patients with advanced cancer do not receive sufficient symptoms management, but also not get enough attention on their psychological symptoms and not obtain good mental health services. International data also demonstrate that only a small proportion of cancer patients with psychological symptoms receive appropriate treatment.^28^, ^29^ Inadequate treatment of psychological symptoms may be due to clinicians' insufficient understanding of harm of anxiety or depression and their greater emphasis on physical symptoms rather than psychological symptoms. In addition, current study found the symptom burden were associated with disease stage in advanced cancer; therefore, there should be more attention on symptoms management in palliative care.

Several underlying mechanisms may lead to the fact that anxiety and depression were associated with symptom burden in cancer patients. Anxiety and depression may result in abnormal hyperactivity HPA axis, elevated cortisol levels, release of inflammatory markers, less healthy lifestyles and behavior, which lead to multiple physical symptoms, and even a higher cancer‐specific mortality risk for variety types of cancer including CRC.^30^ Some studies suggest that the increase in the proportion of physical symptoms in patients with depression may be due to somatosensory amplification, which indicates that depression can affect the perception and evaluation of physical symptoms through their impact on higher cognitive processing patterns.^11^ Although somatosensory amplification is a possible cause for increased proportion, the physical symptoms of patients are actually occurring and painful. Anxiety and depression increase symptom burden and even lead to poor survival, which should attract the attention of all medical personnel providing services for cancer patients.

Our study found that age and gender were not associated with anxiety, depression and symptom burden in patients with advanced CRC. Previous studies showed that the significance of age and sex in the emergence of anxiety or depression in patients with CRC still remains controversial.^4^ Lloyd et al. found that older and female patients with CRC were more associated with anxiety and depression.^3^ However, Howren et al. showed that younger patients with CRC were more associated with depression compared to older patients.^31^ Mols et al. showed that being male was associated with more depressive symptoms.^5^ Our findings differed from previous studies because these studies mainly recruit early stage patients, but our study recruited patients with advanced cancer. The finding that age and gender were not related to symptom burden was consistent with previous studies.^32^

### 4.1 Strengths and limitations

The strengths of the current study include the data were recruited from 10 different cancer centers with diverse geographical location and level of economic development across China, that increase the generalizability and representativeness of the results.

This study has several limitations. First, we provided assessments for patients classified as anxious or depressed only according to the HADS. This instrument is widely used and is a valid measurement of anxiety and depression in cancer patients. Second, socioeconomic factors that may interfere (e.g., employment status) were not considered. Third, the associations found in our research do not necessarily mean causation. Fourth, previous psychiatric conditions of patients were not evaluated in our study due to the cross‐sectional design. We also did not investigate the mental health problems of patients before they had cancer, but focused on the relationship between their current psychological state and symptom burden. Finally, the exclusion of outpatient patients represents a selection bias in this study. Future research could include advanced‐stage outpatients to explore the differences in symptom burden between outpatients and inpatients.

## 5 CONCLUSION AND IMPLICATION

In conclusion, our findings are instructive as this is the first study to examine the association between anxiety, depression, and symptom burden in advanced CRC patients. Anxiety and depression are associated with a significantly higher symptom burden in patients with advanced cancer. The findings may advance our understanding of the psychological symptoms of advanced CRC patients. We emphasize anxiety and depression as important risk factors related to symptom burden in advanced CRC patients despite the limitations of our study. We hope that the findings of this study can enable oncology healthcare professionals to pay more attention to unrecognized and untreated psychological symptoms in advanced cancer patients and timely identify and handle these symptoms through routine screening and referral in future clinical practice. The implementation of psychological services may help to improve symptom management in patients with advanced cancer in oncology and palliative care.

## AUTHOR CONTRIBUTIONS

**Lili Song:** Conceptualization (equal); formal analysis (equal); writing – original draft (equal); writing – review and editing (equal). **Zhongge Su:** Conceptualization (equal); formal analysis (equal); writing – original draft (equal); writing – review and editing (equal). **Ying Pang:** Conceptualization (equal). **Yi He:** Conceptualization (equal). **Yuhe Zhou:** Data curation (equal). **Yu Wang:** Investigation (equal). **Yongkui Lu:** Investigation (equal). **Yu Jiang:** Investigation (equal). **Xinkun Han:** Investigation (equal). **Lihua Song:** Investigation (equal). **Liping Wang:** Investigation (equal). **Zimeng Li:** Investigation (equal). **Xiaojun Lv:** Investigation (equal). **Yan Wang:** Investigation (equal). **Juntao Yao:** Investigation (equal). **Xiaohong Liu:** Investigation (equal). **Xiaoyi Zhou:** Investigation (equal). **Shuangzhi He:** Investigation (equal). **Yening Zhang:** Investigation (equal). **Jinjiang Li:** Investigation (equal). **Bingmei Wang:** Investigation (equal). **Lili Tang:** Conceptualization (equal); funding acquisition (equal); project administration (equal).

## FUNDING INFORMATION

The author(s) disclosed receipt of the following financial support for the research, authorship, and/or publication of this article: This study was funded by the Scientific and Technological Innovation Project of China Academy of Chinese Medical Sciences (CI2021A01819); and the Scientific Research and Cultivation Program Foundation of Beijing Hospitals Authority, Beijing, China (No. PX2021045).

## CONFLICT OF INTEREST STATEMENT

The author(s) declared no potential conflicts of interest with respect to the research, authorship, and/or publication of this article.

## Data Availability

The data that support the findings of this study are available from the corresponding author upon reasonable request.
